# Cyberbullying a modern form of bullying: let’s talk about this health and social problem

**DOI:** 10.1186/s13052-018-0446-4

**Published:** 2018-01-17

**Authors:** Pietro Ferrara, Francesca Ianniello, Alberto Villani, Giovanni Corsello

**Affiliations:** 1 Institute of Pediatrics, Catholic University Medical School, Rome, Italy; 2 Service of Pediatrics, Campus Bio-Medico University, Rome, Italy; 3 General Pediatrics and Pediatric Infectious Diseases Unit, Bambino Gesù Children’s Hospital, IRCCS, Rome, Italy; 4 Institute of Pediatrics, University of Palermo, Palermo, Italy

**Keywords:** Cyberbullying, Victimization, Children

## Abstract

Cyberbullying or electronic aggression has already been designated as a serious public health threat. Cyberbullying should also be considered as a cause for new onset psychological symptoms, somatic symptoms of unclear etiology or a drop in academic performance. Pediatricians should be trained to play a major role in caring for and supporting the social and developmental well-being of children.

Cyberbullying or electronic aggression has already been designated as a serious public health threat and elicited warnings to the general public from the Centers for Disease Control and Prevention (CDC) [1]. The term appears to have been coined in 2000 in Canada by the owner of a Web site dedicated to preventing traditional (face-to-face) bullying [1]. Tokunaga defined the phenomenon as “any behavior performed through electronic or digital media by individuals or groups that repeatedly communicates hostile or aggressive messages intended to inflict harm or discomfort on others” [2]. This definition highlights several important cyberbullying features: the technology component, the hostile nature of the act, the intent to cause suffering, considered by most scholars to be crucial to the definition, and repetitiveness [1].

Common forms of cyberbullying involve mobile phones (bullying by phone calls, text messages, picture/video clip bullying including so-called ‘happy slapping’) or using the internet (bullying by emails, chat room, through instant messaging and via websites, including blogs) [3, 4].

Some twenty-first century factors have contributed to making cyberbullying a public health concern: the increasing penetration of networked computers and mobile phones among young people, the advent of social media and the reliance on new connectivity tools to the point where many would rather tolerate negative effects than be disconnected [1].

Young people today have direct access to the Internet from personal computers and mobile devices, whether at home, schools or in public places. Reports from 2012 indicate that 95% of American teenagers use the Internet and, of those, 81% use social media [1]. As early as 2009, polls showed that more than half of adolescents were logging on to a social media website more than once per day and that 22% logged on to a preferred website more than 10 times per day [5]. Computer time accounts for up to 1.5 h per day; half of this is spent in social networking, playing games, or viewing videos [6].

Adolescents are connected to social media at a time when their levels of social and emotional development leave them vulnerable to peer pressure and when they have a limited capacity to self-regulate [5].

Cyberbullying victims may underreport, too, for fear their parents will restrict their time on the Internet/cell phones or discover information that the adolescents themselves have posted on the web, for fear of punishment by the bully or for embarrassment about being perceived as weak [1, 7].

Cyberbullying have its own identifying characteristics, which include: the possible anonymity of the bully, the larger potential audience for the abuse being carried out, the difficulty of disconnecting oneself from the cyber environment and the absence of the direct face to face contact which is present in many types of traditional bullying [4, 8].

Anonymity, by promoting disinhibition, can lead to magnified aggression because the perpetrator may feel out of reach and immune to retribution; moreover the ability to hide behind fake screen names, or using someone else’s screen name, provides bullies with the opportunity to communicate things they would be reticent to say to another’s face, without see the target’s emotional reactions and realize that their comments have been carried too far or misinterpreted [1, 5, 7].

Some adolescents mistake cyberbullying as making fun of peers and suddenly realizing the severity of the situation after the effects have already snowballed.

The motivation behind cyberbullying, as reported by both cyberbullies and non-bullies, included a lack of confidence or the desire to feel better about themselves, a desire for control, finding it entertaining and retaliation [4, 5].

Research reveals that individuals who are victims of cyberbullying are targets of traditional bullying as well, but in traditional accounts of bullying, the aggressive behaviors generally occur during school hours and cease once victims return home [2]. Cyberbullying, in contrast, is far more pervasive in the lives of those who are victimized, because they can be reached at any given time of the day, therefore the persistence of the bullying behaviors may result in even stronger negative outcomes than traditional bullying [2, 5].

Moreover, cyberbullying has the same risk factors found in traditional bullying but involves others which should not be overlooked, such as the little control exerted over personal information, which may result from ignorance about the risks of sharing personal information on Internet, sharing passwords, communicating with strangers, openly displaying very personal information such as addresses and telephone numbers [8].

Rather than physically stronger, cyberbullies tend to be more technologically savvy and better able to access victims online, hide their electronic trails, and take advantage of the expanded bullying “repertoire”, which now includes identity theft, account hacking, infecting a victim’s computers, impersonation, or posting embarrassing content [1].

Relationships have also been discovered between cyberbullying and Internet addiction, the latter being understood as a continuous urge to connect to Internet which restricts forms of entertainment and social relationships, seriously affects an individual’s moods and irritability, induces violent, aggressive behavior that makes it impossible to disconnect and increases the user’s own social isolation and the destruction of their own closest relationships [8].

All adverse childhood experiences, typically defined as stressful or traumatic life events that occur during the first years of life, are pervasive and notable public health problems [9]. Cyberbullying should also be considered as a cause for new onset psychological symptoms, somatic symptoms of unclear etiology or a drop in academic performance [1]. Victims of cyberbullying have lower self-esteem, higher levels of depression, behavioral problems, substance abuse and experience significant life challenges [2, 5]. Moreover, bullying victimization may trigger a sequence of events that results in suicidal behavior; Ferrara et al. identified in Italy 55 cases of suicide among children and young adults <18-year-old between January 2011 and December 2013 and 4 (7.3%) were bullying victims [10]. After several suicides were linked to cyberbullying, media attention to such cases gradually increased, becoming more intense in recent years [1].

In Italy, ISTAT data published in 2015 shows that, among the mobile and/or Internet users ages 11–17, the 5.9% report being bullied via SMS, email, chat or social networks; Police data report that 235 cases of cyberbullying have been reported in 2016 [11, 12].

Recently Law no. 71/17, the so-called “anti-cyberbullying law”, officially entered into force after being approved by the Italian Parliament, intended to tackle online bullying of children after several high-profile cases in which victims have committed suicide [13]. The legislation provides a specific legal definition of cyberbullying for the first time in Italy, and requires all schools to educate pupils to use the Internet responsibly and to have a member of staff responsible for tackling the problem. It makes it illegal to use the Internet to offend, slander, threaten or steal the identity of a minor, and allows the victim or their parent to demand that websites hosting abusive content remove it within 48 h.

A major practical step is to increase awareness among adults; many adults of the current parental generation are not aware of the varied potential of mobile phones and the internet, to the same extent as young people [4]. Parents of seventh or eighth graders should be made aware of their child’s potential victimization and ways they can open and maintain communication to prevent or remedy such incidences [2]. Training should be provided to junior high school teachers, counselors, and school administrators for the detection and remediation of this social problem [2, 4].

Pediatricians should be trained to play a major role in caring for and supporting the social and developmental well-being of children raised in variously conditions and in new types of problems [9, 14, 15]. A strong case can be made for introducing screening questions about children’s online lives into the general pediatric visit, including queries about excessive video game use and cyberbullying [1].

Guidelines can provide a useful framework for all concerned to reduce cyberbullying and its negative effects and are now becoming available in many countries to assist parents, young people and schools to understand the problem and take effective action.

The strategy is to provide information for youth, parents, and school personnel on what is cyberbullying and how to avoid being a victim, trough lessons, interactive computer game, forum, websites, tip sheets and other online resources; interesting programs to decreasing cyberbullying and cyber victimization exist, but much more research is needed to understand the long-term impact of these interventions [16, 17].

## References

[1] Aboujaoude E, Savage MW, Starcevic V, Salame WO (2015). Cyberbullying: review of an old problem gone viral. J Adolesc Health.

[2] Tokunaga (2010). Following you home from school: a critical review and synthesis of research on cyberbullying victimization. Comput Hum Behav.

[3] Centers for Disease Control and Prevention. Youth violence: technology and youth protecting your child from electronic aggression; 2014. http://www.cdc.gov/violenceprevention/pdf/ea-tipsheet-a.pdf. Accessed 11 September 2017.

[4] Smith PK, Mahdavi J, Carvalho M, Fisher S, Russell S, Tippett N (2008). Cyberbullying: its nature and impact in secondary school pupils. J Child Psychol Psychiatry.

[5] Hamm MP, Newton AS, Chisholm A, Shulhan J, Milne A, Sundar P (2015). Prevalence and effect of cyberbullying on children and young people: a scoping review of social media studies. JAMA Pediatr.

[6] Council on communications and media (2013). Children, adolescents, and the media. Pediatrics.

[7] Kowalski RM, Limber SP (2007). Electronic bullying among middle school students. J Adolesc Health.

[8] Casas JA, Del Rey R, Ortega-Ruiz R (2013). Bullying and cyberbullying: convergent and divergent predictor variables. Comput Hum Behav.

[9] Ferrara P, Bernasconi S (2017). From "classic" child abuse and neglect to the new era of maltreatment. Ital J Pediatr.

[10] Ferrara P, Ianniello F, Cutrona C, Quintarelli F, Vena F, Del Volgo V (2014). A focus on recent cases of suicides among Italian children and adolescents and a review of literature. Ital J Pediatr.

[11] Il bullismo in Italia: comportamenti offensivi e violenti tra i giovanissimi. http://www.istat.it/it/files/2015/12/Bullismo.pdf?title=Bullismo++tra. Accessed 28 November 2017.

[12] Commissariato di PS, Una vita da social. https://www.commissariatodips.it/uploads/media/Comunicato_stampa_Una_vita_da_social_4__edizione_2017.pdf. Accessed 28 November 2017.

[13] Law n. 71/17 of 29/05/2017, GU n. 127 of 03/06/2017. Senato della Repubblica. http://www.senato.it/leg/17/BGT/Schede/Ddliter/43814.htm. Accessed 11 September 2017.

[14] Ferrara P, Corsello G, Basile MC, Nigri L, Campanozzi A, Ehrich J, Pettoello-Mantovani M (2015). The economic burden of child maltreatment in high income countries. J Ped.

[15] Ferrara P, Vena F, Caporale O, Del Volgo V, Liberatore P, Chiaretti A, Riccardi R (2013). Children left unattended in parked vehicles: a focus on recent italian cases and a review of literature. It. J Ped.

[16] Espelage DL, Hong JS. Cyberbullying prevention and intervention efforts: current knowledge and future directions. Can J Psychiatr. 2017 Jun;62(6):374–80. 10.1177/0706743716684793. Epub 2016 Dec 19 10.1177/0706743716684793 PMC5455869 28562094

[17] Del Rey R, Casas JA, Ortega R. Impact of the ConRed program on different cyberbulling roles. Aggress Behav. 2016 Mar-Apr;42(2):123–35. 10.1002/ab.21608. Epub 2015 Sep 9 10.1002/ab.21608 26351131

